# Impact of Time of Administration, Fasting, and a Low-Carbohydrate Diet on Alpelisib-Associated Hyperglycemia and Efficacy: A Pilot Randomized Controlled Phase IIb Trial

**DOI:** 10.3390/cancers18071156

**Published:** 2026-04-03

**Authors:** Eduard Vrdoljak, Marija Pancirov, Josipa Flam, Dora Čerina Pavlinović, Matea Jakas Vučić, Marica Barać, Natalija Dedić Plavetić, Paula Podolski, Mladen Krnić, Žarko Bajić

**Affiliations:** 1 Department of Oncology, University Hospital Center Split, Spinčićeva ul. 5, 21000 Split, Croatia; dora09cerina@gmail.com (D.Č.P.); matea.jakas@gmail.com (M.J.V.); maricabarac1@gmail.com (M.B.); 2 School of Medicine, University of Split, Šoltanska ul. 2, 21000 Split, Croatia; mladen.krnic@gmail.com; 3 Ordinacija za Bolesti Dojke, Ordinacija dr. Vedrana Buljević, Spinčićeva 2, 21000 Split, Croatia; jazvo.marija@gmail.com; 4 Department of Radiotherapy and Oncology, University Hospital Center Osijek, Ul. Josipa Huttlera 4, 31000 Osijek, Croatia; jflam@mefos.hr; 5 School of Medicine, Josip Juraj Strossmayer University of Osijek, Ul. Josipa Huttlera 4, 31000 Osijek, Croatia; 6 Department of Oncology, University Hospital Center Zagreb, Ulica Mije Kišpatića 12, 10000 Zagreb, Croatia; ndedic@kbc-zagreb.hr (N.D.P.); podolski.paula@gmail.com (P.P.); 7 School of Medicine, University of Zagreb, Šalata 2, 10000 Zagreb, Croatia; 8 Department of Endocrinology, University Hospital Center Split, Spinčićeva 1, 21000 Split, Croatia; 9 Research Unit “Dr. Mirko Grmek”, Psychiatric Clinic Sveti Ivan, Jankomir 11, 10090 Zagreb, Croatia; zarko@biometrika.hr; 10 Biometrika Healthcare Research, Buzinski prilaz 1, 10010 Zagreb, Croatia

**Keywords:** breast cancer, breast neoplasms, *PIK3CA* mutation, hyperglycemia, alpelisib, diet, glucose, fulvestrant

## Abstract

***Background***: Alpelisib plus fulvestrant improves outcomes in *PIK3CA*-mutated, hormone receptor-positive, HER2-negative metastatic breast cancer. However, on-target hyperglycemia often leads to dose modification or discontinuation. We aimed primarily to determine whether evening alpelisib after a ≥5 h fast with low-carbohydrate guidance reduces severe hyperglycemia versus standard morning dosing, and secondarily, to assess time to first grade 3–4 hyperglycemia, efficacy, and quality of life (QoL). ***Methods***: ITACA was an open-label, randomized, phase IIb trial in three Croatian centers. Patients progressing on endocrine therapy were randomized 1:1 to evening alpelisib 300 mg after a ≥5 h fast with low-carbohydrate guidance or standard morning alpelisib, both with fulvestrant. The primary endpoint was the exposure-adjusted incidence rate (EAIR) of first grade 3–4 hyperglycemia within 90 days or 30 days post-discontinuation. Secondary endpoints were time to first grade 3–4 hyperglycemia, efficacy, and QoL. ***Results***: Forty-two patients were randomized (21 per arm). Median age was 60 vs. 63 years in the evening vs. morning arms. In the safety set, EAIR of first grade 3–4 hyperglycemia was 378 vs. 742 per 100 person-years (11/21 vs. 14/20 patients with ≥1 event, unadjusted IRR 0.51, 95% CI 0.23–1.12). Adjusted Poisson models favored evening dosing. Analyses suggested delayed onset (median 73 vs. 9.5 days), with no detriment in efficacy or QoL. ***Conclusions***: Evening alpelisib preceded by fasting and low-carbohydrate guidance may improve metabolic tolerability without compromising efficacy or QoL. These findings support evaluation in a larger trial incorporating prospective metabolic adherence and pharmacokinetic assessments.

## Simple Summary

Alpelisib is an important targeted treatment for patients with advanced hormone receptor-positive, HER2-negative breast cancer, but its clinical use is often limited by treatment-related hyperglycemia. This metabolic side effect frequently leads to dose reductions or treatment discontinuation. Because alpelisib directly interferes with normal insulin signaling, the metabolic conditions at the time of drug administration may influence the severity of hyperglycemia. In this randomized pilot study, we investigated whether evening administration of alpelisib after a short fasting period, combined with low-carbohydrate dietary guidance, could improve metabolic tolerability compared with standard morning dosing. Our findings indicate that this strategy may reduce the frequency and delay the onset of severe hyperglycemia without negatively affecting treatment efficacy or quality of life. These results suggest that simple, behavior-based interventions may optimize the therapeutic use of alpelisib and warrant further evaluation in larger clinical trials.

## 1. Introduction

Breast cancer (BC) is the most commonly diagnosed malignancy in women worldwide and remains a leading cause of cancer death [1]. Approximately 70% of invasive BC are hormone receptor-positive (HR-positive) and HER2-negative, making HR-positive/HER2-negative disease by far the most common molecular subtype [2], where endocrine therapy is the backbone of systemic treatment, increasingly combined with targeted agents [3,4,5,6,7]. In this setting, activating mutations in the phosphatidylinositol-4,5-bisphosphate 3-kinase catalytic subunit alpha (PIK3CA) pathway occur in a substantial proportion of tumors and are associated with endocrine resistance, establishing the PI3Kα pathway as an important therapeutic target [8,9].

Alpelisib, an oral PI3Kα-selective inhibitor, combined with fulvestrant, has been shown to prolong progression-free survival (PFS) in patients with *PIK3CA*-mutated HR-positive, HER2-negative metastatic BC progressing on prior endocrine therapy, and is now one of the standard treatment options in this patient population [10,11,12]. Fulvestrant is an intramuscular selective estrogen receptor degrader (SERD) that binds to and accelerates degradation of estrogen receptors, thereby inhibiting estrogen-driven signaling in hormone receptor-positive breast cancer [13]. The clinical utility of alpelisib is limited by a distinctive toxicity profile dominated by on-target hyperglycemia [14,15]. In pivotal trials, hyperglycemia was common, often severe, and typically occurred within the first weeks of treatment, leading to dose reductions, interruptions, and discontinuations, thereby potentially compromising efficacy outcomes [10,14]. Through its mode of action, alpelisib-induced hyperglycemia results from inhibition of insulin-mediated PI3K signaling, reducing glucose uptake in skeletal muscle and adipose tissue and increasing hepatic glucose output with compensatory hyperinsulinemia. This insulin surge may, in turn, reactivate PI3K–mTOR signaling in tumor cells, potentially attenuating antitumor efficacy [16]. Preclinical and clinical pharmacology data suggest that the metabolic and nutritional context at the time of dosing—including dosing time and reduced carbohydrate intake—can modulate both the glycemic impact and the pharmacodynamic profile of PI3K inhibition [17]. To our knowledge, no randomized trial has specifically tested whether modifying the timing and metabolic context of alpelisib administration can reduce treatment-emergent hyperglycemia while preserving efficacy and quality of life. This question is clinically relevant because severe hyperglycemia is one of the main barriers to the optimal use of alpelisib in routine practice [18].

The aim of our study was to test a pragmatic strategy to reduce alpelisib-related hyperglycemia by aligning dosing with an evening schedule after a short fasting window and providing low-carbohydrate dietary guidance. We compared this approach with standard morning dosing, with the primary objective of assessing the person-time standardized rate of the first occurrence of grade 3–4 hyperglycemia within 90 days or 30 days after discontinuation, whichever occurred first. Secondary objectives were to assess differences in time to first occurrence of grade 3–4 hyperglycemia, efficacy, and quality of life (QoL), which is, besides overall survival, a key treatment goal in patients with PIK3CA-mutated HR-positive, HER2-negative metastatic breast cancer, who often receive multiple lines of therapy and experience cumulative toxicities [19]. In the ITACA study, we therefore prospectively evaluated HRQoL to determine how treatment-emergent toxicities, particularly alpelisib-related hyperglycemia, affect patients’ daily functioning and overall well-being.

## 2. Methods

### 2.1. Trial Design

Randomized, open-label, parallel-group phase IIb trial conducted at three Croatian university hospitals (Split, Zagreb, Osijek) between September 2022 and February 2025. The protocol was approved by the Central Ethics Committee of HALMED (Središnje etičko povjerenstvo HALMED; approval code 530-07/21/29; approval date: 30 September 2021). All patient data were pseudonymized, with General Data Protection Regulation (GDPR)-compliant handling and separation of identifiers from clinical data. All patients provided written informed consent. The study was conducted in accordance with the Declaration of Helsinki. Study protocol was registered at EudraCT 2021-000845-42. The trial record was first entered in the EudraCT database on 16 May 2022. Patients and the public were not involved in the design, conduct, reporting, or dissemination plans of this trial.

### 2.2. Sample Size Determination

Sample size was considered for the primary safety endpoint of grade 3–4 hyperglycemia. Using SOLAR-1 as an external benchmark, in which grade 3–4 hyperglycemia occurred in 36.6% of patients treated with alpelisib plus fulvestrant, a conventional two-sided comparison with α = 0.05 and 80% power to detect a reduction to 23.6% would have required 676 patients (338 per arm) under a simple proportion-based framework. Because such a trial was not feasible, ITACA was designed as a pilot phase IIb study with a pragmatic target sample size of 60 patients (30 per arm), intended to provide preliminary safety, feasibility, and effect-size estimates rather than definitive confirmatory evidence.

### 2.3. Changes After Trial Commencement

After 42 patients had been recruited, we performed a blinded reassessment of sample size requirements using the observed incidence of grade 3–4 hyperglycemia and the empirical distribution of key baseline covariates. This indicated that a reliably powered comparison would still require several hundred participants and that increasing the sample from 42 to the originally planned 60 would have had a negligible impact on statistical power. In view of this, together with slow accrual and finite resources, recruitment was stopped at 42 patients and the study is reported as exploratory and hypothesis-generating. No other protocol-level changes to endpoints, analysis populations, primary hypothesis testing, or statistical methods were made before outcome data for the randomized comparison became available.

No formal interim efficacy analyses or prespecified stopping rules were used.

### 2.4. Study Population

Eligible patients were postmenopausal women with HR-positive, HER2-negative, PIK3CA-mutated metastatic breast cancer with documented progression on endocrine therapy, typically after or with a CDK4/6 inhibitor. Key eligibility criteria included age ≥ 18 years, ECOG performance status 0–1, adequate organ function, measurable disease or lytic bone lesions, and prespecified screening glycemic thresholds (fasting plasma glucose ≤ 140 mg/dL, HbA1c ≤ 6.4%). Key exclusions included type 1 diabetes or uncontrolled type 2 diabetes, clinically significant or uncontrolled comorbidities, active or unstable CNS metastases, recent major surgery, and concomitant strong CYP3A modulators or systemic corticosteroids without washout.

### 2.5. Intervention

All patients received fulvestrant 500 mg intramuscularly on Day 1 and Day 15 of Cycle 1 and every 28 days thereafter. Experimental arm: alpelisib 300 mg once daily at 22:00 after a ≥5 h fast with low-carbohydrate dietary guidance. Patients randomized to the experimental arm received structured counseling and a written dietary brochure from the study team on a pragmatic low-carbohydrate, low-glycemic-index dietary approach. In the protocol, the low-carbohydrate concept was based on carbohydrate restriction to <130 g/day, but in clinical implementation, the intervention was delivered as pragmatic dietary counseling rather than as a strictly prescribed gram-based diet [20]. Patients were advised to avoid carbohydrate-rich foods such as sugar, bread, pasta, and similar foods, and to preferentially choose lower-carbohydrate foods and protein- or fat-rich options, such as meat, fish, eggs, cheese, nuts, and non-starchy vegetables. Alpelisib in the experimental arm was taken at 22:00, at least 5 h after the last meal. Immediately before dosing, patients were instructed to consume either 200 g yogurt, 30–50 g almonds, or 100 g of semi-hard cheese with water, after which they were advised not to eat again that night. The control arm received alpelisib 300 mg once daily in the morning per approved prescribing information. Treatment continued until progression, unacceptable toxicity, death, or withdrawal. Hyperglycemia was managed per standard practice, including antihyperglycemic therapy and dose modification when indicated. No pharmacokinetic sampling was performed [21].

### 2.6. Randomization

Patients were randomized 1:1 using minimization with equal weights for eight baseline factors (age, center, metastasis pattern, prior CDK4/6 inhibitor, prior chemotherapy, baseline HbA1c, baseline antidiabetic therapy, and BMI). Allocation was performed centrally by an independent biostatistician. The personnel enrolling participants did not have access to the random allocation sequence. This was an open-label trial, and participants, treating clinicians, and investigators were not blinded after assignment to intervention.

### 2.7. The Primary Outcome

The primary endpoint was the treatment-emergent exposure-adjusted incidence rate (EAIR) of first grade 3–4 hyperglycemia within 3 months of treatment or 30 days after permanent discontinuation, whichever occurred first. EAIR was calculated as (patients with ≥1 grade 3–4 event ÷ person-years at risk) × 100. Glycemic assessment included fasting plasma glucose and HbA1c at screening and scheduled visits, plus home glucose monitoring. Patients completed a 7-point capillary profile for three consecutive days before the first dose and repeated it starting three days after initiation. Thereafter, capillary glucose was measured twice daily, timed before evening dosing in the experimental arm and at bedtime in the control arm. Hyperglycemia was graded per CTCAE v4.03 based on continuous glucose monitoring (FreeStyle Libre; Abbott Diabetes Care, Alameda, CA, USA). Grade 3–4 hyperglycemia was defined as the first reading meeting CTCAE grade 3 or 4 criteria within the risk window. Implausible isolated sensor readings were reviewed against contemporaneous capillary measurements when available.

### 2.8. Secondary Outcomes

Secondary outcomes were (1) time to first occurrence of grade 3–4 hyperglycemia in days from Day 2 after the alpelisib initiation; (2) objective response rate; (3) PFS; and (4) QoL.

### 2.9. Statistical Analysis

We defined two analysis populations. The intention-to-treat (ITT) population included all randomized patients and was used for tumor efficacy endpoints. The modified ITT (mITT, “safety set”) included all randomized patients who received at least one dose of alpelisib and was used for safety, glycemic, and quality-of-life analyses. Missing data were not imputed. Time-to-event endpoints were analyzed with right-censoring, and other endpoints were analyzed on a complete-case basis.

For the primary endpoint, EAIR was calculated in each arm as (patients with at least one grade 3–4 event within the prespecified risk window divided by total person-years at risk) multiplied by 100, counting only the first qualifying event per participant. Between-arm comparisons used Poisson regression with log link and an offset for log(person-time), reporting incidence rate ratios (IRRs) with robust standard errors and two-sided 95% confidence intervals. Adjusted Poisson models included the protocol-specified minimization factors as covariates, and a Day 2 landmark sensitivity analysis was applied to address between-arm differences in initial exposure timing.

Time to first grade 3–4 hyperglycemia (hyperglycemia-free survival) in the mITT population was analyzed using Kaplan–Meier methods and Cox proportional hazards models with time zero defined as Day 2. Participants without an event were censored at the last glucose assessment within the risk window. Cox models reported hazard ratios (HRs) with two-sided 95% confidence intervals, unadjusted and adjusted for the same covariate set.

Objective response rate (ORR) in the ITT population was summarized with exact binomial 95% confidence intervals. Progression-free survival (PFS) was defined from randomization to radiographic progression or death, and analyzed using Kaplan–Meier curves, log-rank tests, and Cox models adjusted for the base covariate set.

EORTC QLQ-C30 and QLQ-BR23 were scored per EORTC manuals (0–100 scales) and compared at approximately three months using ANCOVA with baseline score adjustment, reporting adjusted mean differences (experimental minus control) with two-sided 95% confidence intervals and *p* values. To limit false positives across prespecified families of analyses, we controlled the false discovery rate at 5% using the Benjamini–Hochberg procedure.

All tests were two-sided with nominal α = 0.05. Analyses were performed in Stata 19 (StataCorp LLC, College Station, TX, USA).

## 3. Results

### 3.1. Participant Characteristics

**Figure 1:**
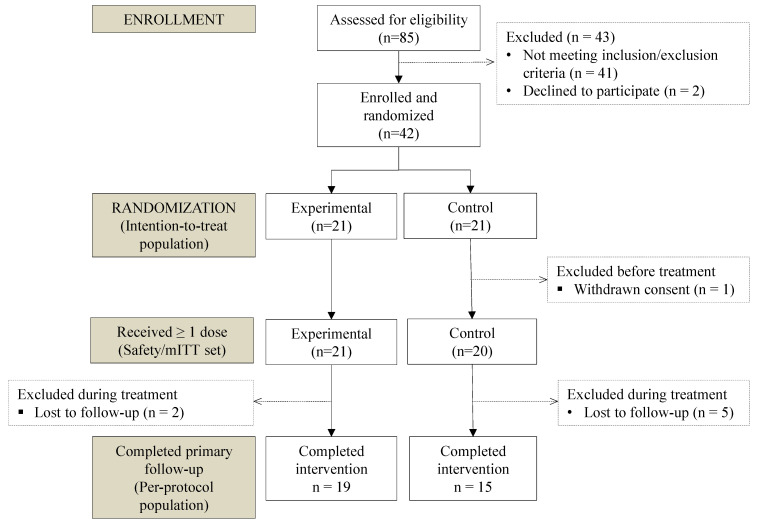
CONSORT flow diagram of screening, exclusions, randomization, treatment exposure, and analysis sets; mITT, modified intention-to-treat.

Participant flow through screening, exclusions, randomization, treatment exposure, and analysis sets is shown in Figure 1. Between 17 September 2022 and 28 February 2025, 42 patients were randomized (21 per arm), of whom 41 received ≥1 alpelisib dose (mITT) (Figure 1). Baseline characteristics were broadly comparable between arms (Table 1). Median age at randomization was 60 years (IQR 54–68) in the experimental group and 63 (57–72) in the control. Median body mass index (BMI) was higher in the experimental arm, but the number of obese participants was identical. ECOG performance status favored the experimental arm. Prior hyperglycemia requiring treatment was rare and similar in both groups. Time from the last documented progression to alpelisib initiation was short in both arms, with a longer median in the control than in the experimental arm (Table 2). Disease distribution was predominantly mixed bone–visceral in both arms, with bone and liver among the most commonly involved sites. Lung involvement was similar in both groups, and brain metastases were rare.

*PIK3CA* status showed expected heterogeneity, with unknown results more frequent in the experimental arm (37.5% vs. 5.6%), H1047X more common in the control, and E545K observed only in the control, whereas E542K was present in both groups. Hormone receptors were strongly expressed at progression: ER positivity was universal (100.0% in both arms) with high median ER percentages (90% vs. 95%); PR positivity was frequent with higher median PR in the experimental arm (80% vs. 60%). Proliferation indices were similar, and high Ki-67 (≥20%) predominated in both arms (70.6% vs. 57.9%). These patterns indicate broadly comparable tumor biology at the last progression, with modest imbalances in mutation subtypes and grade, likely reflecting small-sample variation.

Baseline glycemic parameters were well balanced, with identical median HbA1c (5.6% in both arms) and similar fasting plasma glucose (5.4 vs. 5.1 mmol/L) in the experimental and control arms.

### 3.2. Primary Endpoint

Within the prespecified risk window, the EAIR of the first occurrence of grade 3–4 hyperglycemia was 378 per 100 person-years in the experimental arm and 742 per 100 person-years in the control arm (unadjusted IRR 0.51, 95% CI 0.23–1.12).

As a sensitivity analysis to address a potential Day 1 dosing-time asymmetry, we repeated the EAIR calculation starting from Day 2, thereby excluding Day 1 person-time and any events occurring on that day. This analysis yielded consistent results, with EAIRs of 428 per 100 person-years (95% CI 214–766) in the control arm and 269 per 100 person-years (95% CI 123–511) in the experimental arm. The unadjusted incidence rate ratio (IRR; experimental vs. control) was 0.63 (95% CI 0.26–1.52; *p* = 0.303). It is important to note that the primary EAIR endpoint counts only the first qualifying event per participant within the risk window; subsequent episodes are not included.

After adjustment for the protocol-specified base covariate set (study center, age, location of metastasis, prior chemotherapy, baseline HbA1c, and BMI), the adjusted incidence rate ratio (aIRR) for the experimental versus control arm was 0.37 (95% CI 0.13–1.08; *p* = 0.058) from Day 1 and 0.28 (95% CI 0.10–0.83; *p* = 0.017; FDR < 5%) from Day 2.

We performed additional adjustments for the number of prior treatment lines, baseline fasting plasma glucose (mmol/L), time from relapse to study enrollment, and ECOG performance status. Compared to the control arm, the experimental arm was associated with a statistically significant reduction in the rate of grade 3–4 hyperglycemia (aIRR 0.25; 95% CI 0.07–0.79; *p* = 0.021; FDR < 5%). Exposure-adjusted rates are shown in Table 3, and adjusted Poisson models are shown in Table 4.

**Table 4:** Exposure-adjusted incidence rate ratios for grade 3–4 hyperglycemia (adjusted Poisson model, safety set, mITT).

	aIRR	(95% CI)	*p*
From Day 1			
Treatment arm			
Control	1.00		
Experimental	0.37	(0.13–1.08)	0.058
Study center			
Osijek	1.00		
Split	1.55	(0.12–11.26)	0.706
Zagreb	0.68	(0.18–2.36)	0.561
Age (years)	1.04	(0.99–1.11)	0.16
Location of metastasis			
Bone only	1.00		
Visceral	0.17	(0.01–2.95)	0.232
Both visceral and bone	1.57	(0.33–11.48)	0.604
Prior chemotherapy			
No	1.00		
Yes	0.80	(0.30–2.29)	0.662
Baseline HbA1c (%)	4.38	(1.87–10.56)	0.001
BMI (kg/m^2^)	1.24	(1.07–1.42)	0.003
From Day 2			
Treatment arm			
Control	1.00		
Experimental	0.28	(0.10–0.83)	0.017
Study center			
Osijek	1.00		
Split	7.58	(0.78–42.52)	0.042
Zagreb	2.20	(0.52–8.29)	0.254
Age (years)	1.00	(0.93–1.07)	0.905
Location of metastasis			
Bone only	1.00		
Visceral	0.61	(0.04–8.79)	0.709
Both visceral and bone	2.50	(0.47–19.48)	0.314
Prior chemotherapy			
No	1.00		
Yes	3.46	(0.92–14.10)	0.07
Baseline HbA1c (%)	1.76	(0.81–3.60)	0.128
BMI (kg/m^2^)	1.16	(1.01–1.33)	0.037

Abbreviations: aIRR, adjusted incidence rate ratio estimated using Poisson regression with log link and an offset of log(person-years) using robust (Huber–White) standard errors; CI, two-sided 95% Wald confidence interval; *p*, two-sided Wald *p*-value.

### 3.3. Time to First Occurrence of Grade 3–4 Hyperglycemia

**Figure 2:**
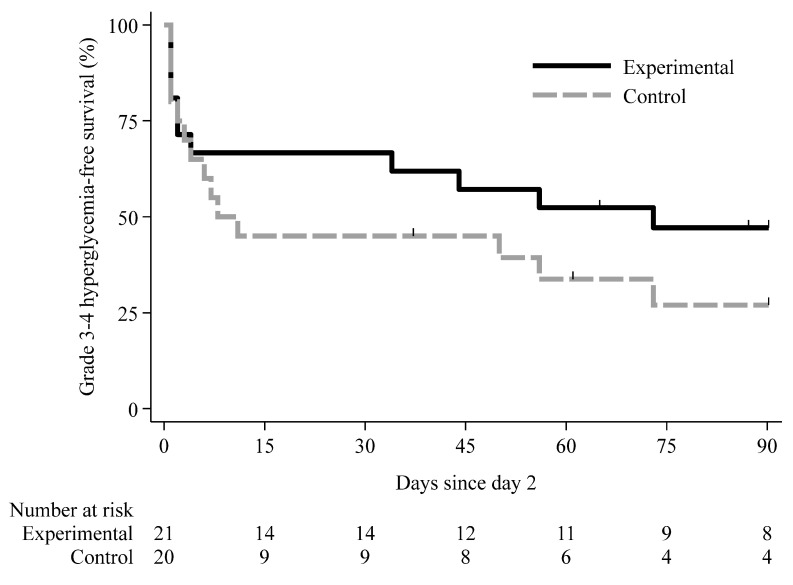
Time to first occurrence of grade 3–4 hyperglycemia in days since Day 2 in the safety set (mITT: received ≥1 dose of alpelisib).

During the prespecified study window (Day 2 through 3 months or 30 days post-discontinuation, whichever occurred first), median grade 3–4 hyperglycemia-free survival (HFS) was 9.5 days in the control arm (95% bootstrap CI 3–73) and 73 days in the experimental arm (95% bootstrap CI 3–73) (Figure 2). Events occurred in 14/20 (70.0%) control and 11/21 (52.4%) experimental group participants during this period. The point estimates suggest longer HFS in the experimental arm, but the wide, overlapping CIs, reflecting small sample size and censoring, indicate substantial uncertainty. On Day 1, five grade 3–4 events were observed (two in the experimental arm and three in the control arm) and were excluded from the Day 2 landmark analysis. In the unadjusted Cox model, the hazard ratio (HR) for the experimental arm versus the control arm was 0.70 (95% CI 0.29–1.68; *p* = 0.422), indicating no statistically significant difference in the hazard of first occurrence of grade 3–4 hyperglycemia. After adjustment for the protocol-specified base covariate set (study center, age, location of metastasis, prior chemotherapy, baseline HbA1c, and BMI), the treatment effect remained non-significant (HR 0.71; 95% CI 0.28–1.78; *p* = 0.466).

### 3.4. Objective Response Rate

In the intention-to-treat population (n = 21 per arm), 10 participants (47.6%) in the experimental arm and eight (38.1%) in the control arm were not evaluable for objective response. A substantial proportion of patients were not evaluable for response due to early discontinuation or missing on-treatment imaging within the response assessment window. Among evaluable patients, the objective response rate (ORR) was 18.2% with the experimental regimen (2/11; all partial responses) and 8.3% with the control (1/12; partial response).

### 3.5. Progression-Free Survival

PFS was analyzed in 42 randomized participants (21 per group) in the intention-to-treat population, with 16 progression or death events observed. Kaplan–Meier estimates suggested longer PFS with the experimental regimen, with 6/21 (28.6%) events versus 10/21 (47.6%) in the control arm (log-rank χ^2^(1) = 2.31; *p* = 0.129). The median PFS was not reached in the experimental group during the observed follow-up (maximum 14.4 months; 95% CI 4.9–not reached), whereas the control group had a median PFS of 8.0 months (95% CI 3.7–11.6). In the multivariable Cox regression adjusted for the base covariate set, the experimental regimen was associated with a significantly lower hazard of progression compared with control (HR 0.20, 95% CI 0.04–0.92; *p* = 0.038; FDR < 5%). This estimate was based on few events and should be interpreted as exploratory.

### 3.6. Quality of Life

Quality-of-life scores at around 3 months were generally comparable between arms. End-of-study QoL data were available for 13 patients in the experimental arm and 10 in the control arm. On the QLQ-C30, there were non-significant trends in favor of the experimental regimen for physical functioning (adjusted mean difference 9.5 points; 95% CI −1.9 to 20.9) and pain (−12.3 points; 95% CI −25.9 to 1.3), while global health status and other domains showed only small, imprecise differences. On the QLQ-BR23, sexual functioning was statistically higher in the experimental arm (7.2 points; 95% CI 0.1 to 14.2), whereas most other functional and symptom scales, including body image and systemic therapy side effects, were close to null. Breast symptoms showed a possible worsening with the experimental strategy (8.8 points; 95% CI −1.6 to 19.2), but with wide confidence intervals. Overall, these exploratory data do not indicate a major quality-of-life penalty with the evening-fasting regimen and suggest a potential advantage for physical and sexual functioning. These findings should be interpreted cautiously given the small sample and wide uncertainty.

## 4. Discussion

In this randomized, phase IIb pilot trial, evening alpelisib dosing after a ≥5 h fast with low-carbohydrate dietary guidance was associated with a numerically lower rate of severe (grade 3–4) hyperglycemia compared with standard morning administration, without an obvious compromise in antitumor activity or QoL. Although the primary unadjusted comparison of EAIRs did not reach conventional statistical significance, adjusted Poisson models consistently favored the experimental regimen. Taken together, these findings support the hypothesis that modifying the timing and metabolic context of alpelisib administration may improve its therapeutic index.

Time-to-event analyses also suggested a later onset of severe hyperglycemia with a shift in median time to first grade 3–4 event from 9.5 to 73 days. Although these estimates were imprecise and based on a small number of events, their direction was consistent with the primary EAIR analysis. The internal consistency between the incidence-rate and time-to-event analyses, including the Day 2 landmark approach used to address the Day 1 dosing asymmetry, supports the interpretation that the observed differences are unlikely to reflect random variation alone.

The magnitude and timing of hyperglycemia observed in the control arm are broadly consistent with pivotal alpelisib studies, in which any-grade hyperglycemia occurred in the majority of patients and severe events typically emerged within the first weeks of therapy, contributing to dose modifications and discontinuations [10,11,12,14]. In this context, the approximately halved EAIR of grade 3–4 hyperglycemia with evening dosing plus fasting, and the shift in median time to first severe event from 9.5 to 73 days, are clinically meaningful, even if imprecisely estimated. The direction and internal consistency of the Poisson and Cox models—including sensitivity analyses from Day 2 to control the Day 1 dosing asymmetry—strengthen the interpretation that the observed differences are not simply random variations in a small sample. Nevertheless, given the small sample size and limited number of events, effect estimates are unstable and should be interpreted primarily in terms of direction and plausibility rather than precision.

More broadly, altered glucose metabolism has long been recognized as a feature of cancer biology [22,23]. In the present study, however, we focus on the more specific and clinically relevant setting of PI3Kα inhibition, alpelisib-induced hyperglycemia, and compensatory hyperinsulinemia [16,24]. The biological rationale for this strategy is supported by mechanistic and translational work showing that PI3Kα inhibition disrupts insulin-mediated glucose uptake and increases hepatic glucose output, driving hyperglycemia and compensatory hyperinsulinemia [16,17]. This feedback can reactivate PI3K–mTOR signaling in tumors and potentially attenuate the antitumor effect of PI3K inhibitors [16]. Taken together, these mechanisms suggest that reducing postprandial glucose and insulin excursions around dosing, through timing and macronutrient modification, could mitigate on-target hyperglycemia and potentially preserve pharmacodynamic pathway suppression. Aligning alpelisib administration with a fasting window, evening administration, and reduced carbohydrate intake is a pragmatic way to blunt postprandial glucose and insulin excursions without adding pharmacologic complexity. Although our data cannot directly confirm effects on insulin dynamics or drug exposure in the absence of pharmacokinetic or hormonal measurements, the observed reduction in severe hyperglycemia is consistent with the hypothesis that metabolic context at dosing modulates on-target toxicity.

From an efficacy perspective, the trial was not powered to detect differences in tumor outcomes, and all efficacy analyses must be viewed as exploratory. Nonetheless, the experimental regimen did not show any consistent signs of harm: ORR among evaluable patients was numerically higher, and multivariable Cox regression suggested a lower hazard of progression with evening dosing, with an adjusted PFS HR of 0.20. This pattern is reassuring given concerns that aggressive glycemic control [16] or altered dosing schedules might compromise antitumor activity. If anything, the combination of maintained dose intensity, lower rates of severe hyperglycemia, and a possible PFS advantage is compatible with the concept that reducing hyperinsulinemic feedback might enhance, rather than weaken, PI3Kα inhibition [16]. However, in view of the small number of events and the wide confidence intervals, these efficacy findings should be interpreted as hypothesis-generating and require confirmation in adequately powered trials.

Patient-reported outcomes provide an additional perspective on the net clinical impact of the intervention. At approximately three months, global QoL and most EORTC QLQ-C30 and QLQ-BR23 domains were similar between arms, with no evidence of a significant quality-of-life trade-off for the evening-fasting strategy. The point estimates for physical functioning and pain were within ranges that have been proposed as clinically meaningful for EORTC QLQ-C30 scales at the group level, although the confidence intervals remain wide [25]. This finding is consistent with prior evidence that overall health-related QoL can be maintained in patients receiving alpelisib-based therapy despite metabolic toxicity management [26]. Trends toward better physical functioning and lower pain scores on the QLQ-C30, and a statistically better sexual functioning score on the QLQ-BR23, suggest that any improvement in metabolic tolerability did not come at the cost of perceived well-being and may, if confirmed, translate into clinically relevant benefits for selected aspects of functioning. Conversely, a possible worsening of breast symptoms in the experimental arm highlights that not all domains move in the same direction.

Current hyperglycemia management algorithms focus mainly on pharmacologic interventions, including metformin as first-line therapy, with escalation to sodium-glucose cotransporter 2 (SGLT2) inhibitors and insulin when needed, alongside dose modifications [15,21,24,27]. Selected patients may also receive other agents such as dipeptidyl peptidase-4 (DPP-4) inhibitors or thiazolidinediones (e.g., pioglitazone). Our results suggest that relatively simple, behaviorally anchored modifications—evening alpelisib administration, a prespecified fasting window, and low-carbohydrate guidance—may add an additional layer of control over severe hyperglycemia. In practice, these strategies could be integrated with existing guidelines, particularly in patients at higher metabolic risk, provided that they are supported by appropriate dietary counseling and glucose monitoring [28]. The randomized design, intensive early glycemic profiling, and consistent direction of effect across multiple analytic approaches are strengths that lend credence to this proposition.

These findings raise the hypothesis that similar timing and dietary strategies could be evaluated with other agents associated with clinically meaningful treatment-emergent hyperglycemia, often accompanied by compensatory hyperinsulinemia, but this requires dedicated prospective testing.

### Study Limitations

This trial has several limitations. First, it was a small, phase IIb pilot study with 42 randomized patients and a limited number of events. All estimates of treatment effect on hyperglycemia, PFS, and QoL are therefore imprecise, with wide CIs, and the study is underpowered for conventional hypothesis testing. Early cessation of enrollment below the planned 60 participants and the prespecified data cut-off (28 February 2025) further curtailed follow-up, resulting in immature PFS and OS data, particularly in the experimental arm, where median PFS was not reached. Accordingly, the observed reduction in the hazard of progression with evening dosing should be interpreted as exploratory and susceptible to both type I and type II error. Second, the open-label design, while unavoidable given the nature of the intervention (dosing time and dietary guidance), introduces potential performance and reporting biases. The primary endpoint relied on objective glucose measurements using standardized procedures, which limits detection bias. However, adherence to the ≥5 h fasting window and low-carbohydrate guidance was not formally quantified, and no food diaries or other formal adherence measures were collected. Therefore, although the intervention was protocol-defined and counseling-based, variability in its implementation across participants could not be assessed. Moreover, no pharmacokinetic or insulin measurements were collected, so any mechanistic explanation linking dosing time and fasting to altered alpelisib exposure or insulin dynamics remains inferential. Although CGM data were collected, we did not prespecify or analyze CGM summary metrics such as time in range (TIR) and time above range (TAR), which might have provided a more granular characterization of glycemic burden. Third, the study population was highly selected: patients were treated at three academic centers in a single country, eligibility criteria excluded those with uncontrolled diabetes, and all participants underwent intensive glycemic monitoring with proactive management. These factors may limit the generalizability of the findings to broader, more comorbid real-world populations and to settings with less frequent monitoring, where the incidence and management of hyperglycemia may differ. Fourth, several secondary and exploratory analyses—including multivariable Poisson and Cox models and multiple QoL domains—were performed in the context of few events and small numbers with end-of-study QoL data (often 11–14 patients per arm). This creates a risk of model overfitting and unstable estimates, despite formal control of the false discovery rate. The QoL findings, and the more extreme adjusted effect estimates for hyperglycemia, should therefore be regarded as hypothesis-generating rather than confirmatory. In addition, QoL was assessed using the legacy BC module QLQ-BR23, which was developed in an earlier treatment era and may not fully capture contemporary, therapy-specific symptom burdens and side-effect profiles operationalized in QLQ-BR45 [29]. Finally, tumor response and progression were assessed by local investigators rather than central blinded review, which may introduce some variability in RECIST-based endpoints, although such misclassification is unlikely to be systematically related to dosing schedule.

## 5. Conclusions

This randomized phase IIb pilot trial suggests that aligning alpelisib administration with an evening dosing schedule after a short fasting period and low-carbohydrate dietary guidance can attenuate severe hyperglycemia and delay its onset, without an obvious compromise in antitumor efficacy or QoL. Although the study was underpowered for definitive conclusions and the estimates are imprecise, the directionally consistent reduction in grade 3–4 hyperglycemia across Poisson and time-to-event models, together with preserved dose intensity and a favorable exploratory PFS, supports the biological premise that the metabolic context at the time of dosing modulates the therapeutic index of PI3Kα inhibition. In the absence of a clear QoL penalty, these data indicate that a simple, behaviorally anchored optimization strategy is feasible and may be clinically useful, particularly for patients at higher metabolic risk. These findings support further evaluation in a larger, adequately powered trial incorporating prospective metabolic adherence and pharmacokinetic assessments.

## Figures and Tables

**Table 1:** Participant characteristics at enrollment, after the most recent progression prior to alpelisib initiation (ITT population).

	Experimental (n = 21)	Control (n = 21)
Age (years), median (IQR)	60 (54–68)	63 (57–72)
Education ^a^		
Secondary school	14 (66.7)	14 (70.0)
University	7 (33.3)	6 (30.0)
Number of children, median (IQR) ^a^	2 (1–2)	2 (1–2)
Body mass index (kg/m^2^), median (IQR)	27.5 (23.5–29.1)	24.9 (21.9–27.9)
Obesity (kg/m^2^ ≥ 30)	4 (19.1)	4 (19.1)
ECOG status 1 ^a^	6 (28.6)	8 (40.0)
History of hyperglycemia requiring treatment ^a^	1 (4.8)	1 (5.0)
Breast cancer in family history	4 (19.1)	5 (25.0)
Other cancer in family history	13 (61.9)	8 (40.0)
Laboratory measures		
Hemoglobin A1c (%), median (IQR)	5.6 (5.3–5.8)	5.6 (5.2–5.9)
Fasting plasma glucose (mmol/L), median (IQR) ^b^	5.4 (4.9–5.9)	5.1 (4.8–5.8)
C-peptide (nmol/L), median (IQR) ^b^	1.14 (0.78–1.72)	1.09 (0.67–1.41)
Treatment of metastatic disease		
Chemotherapy	6 (28.6)	6 (28.6)
Total number of chemotherapy cycles, median (IQR) ^c^	13 (8–23)	24 (11–26)
Endocrine therapy	19 (90.5)	20 (95.2)
Fulvestrant	14 (66.7)	12 (57.1)
Aromatase inhibitors	13 (61.9)	12 (57.1)
Tamoxifen	3 (14.3)	2 (9.5)
Duration of endocrine therapy (months), median (IQR) ^d^	42 (13–73)	18 (10–49)
Targeted therapy	19 (90.5)	20 (95.2)
CDK4/6 inhibitors	19 (90.5)	20 (95.2)
Monoclonal antibodies	2 (9.5)	0 (0.0)
Duration of targeted therapy (months), median (IQR) ^e^	26 (9–48)	14 (10–28)
Number of prior metastatic treatment Lines before alpelisib ^f^		
1	7 (41.2)	10 (55.6)
2	5 (29.4)	3 (16.7)
3	2 (11.8)	3 (16.7)
4	2 (11.8)	1 (5.6)
6	1 (5.9)	1 (5.6)
Best response before alpelisib		
Complete response (CR)	0 (0.0)	0 (0.0)
Partial response (PR)	4 (19.1)	5 (23.8)
Stable disease (SD)	14 (66.7)	10 (47.6)
Progressive disease (PD)	1 (4.8)	5 (23.8)
Not applicable (NA)	2 (9.5)	1 (4.8)

Data are presented as n (%) unless otherwise stated. Abbreviations: IQR, interquartile range; ECOG, Eastern Cooperative Oncology Group. ^a^ Available data for 21 patients in experimental and 20 in control arm. ^b^ Available data for 19 patients in experimental and 20 in control arm. ^c^ Only patients treated with chemotherapy; 6 in each arm. ^d^ Only patients treated with endocrine therapy; 19 in experimental and 20 in control arm. ^e^ Only patients treated with targeted therapy; 19 in experimental and 20 in control arm. ^f^ Available data for 17 patients in experimental and 18 in control arm.

**Table 2:** Tumor characteristics at the most recent progression prior to alpelisib initiation (ITT population).

	Experimental (n = 21)	Control (n = 21)
Days from last progression to alpelisib start, median (IQR)	19 (13–32)	25 (16–33)
Metastasis pattern ^a^		
Visceral	3 (14.3)	2 (10.0)
Bone only	3 (14.3)	2 (10.0)
Both visceral and bone	15 (71.4)	16 (80.0)
Metastasis sites		
Bones	18 (85.7)	18 (85.7)
Liver	14 (66.7)	13 (61.9)
Lung	7 (33.3)	8 (38.1)
Lymph nodes	6 (28.6)	5 (23.8)
Breast	3 (14.3)	1 (4.8)
Peritoneal cavity	1 (4.8)	2 (9.5)
Brain	0 (0.0)	1 (4.8)
Pericardial cavity	0 (0.0)	0 (0.0)
Other	1 (4.8)	1 (4.8)
Predominant histologic type ^b^		
Ductal	12 (75.0)	10 (71.4)
Lobular	2 (12.5)	1 (7.1)
Other	2 (12.5)	3 (21.4)
Immunophenotype ^c^		
Luminal A	4 (25.0)	7 (35.0)
Luminal B	12 (75.0)	13 (65.0)
Grade^d^		
Unknown	8 (40.0)	5 (26.3)
1	1 (5.0)	2 (10.5)
2	9 (45.0)	11 (57.9)
3	2 (10.0)	1 (5.3)
Lymphovascular invasion	4 (19.1)	4 (19.1)
Perineural invasion	0 (0.0)	1 (4.8)
*PIK3CA* mutation ^e^		
Unknown	6 (37.5)	1 (5.6)
E542K	3 (18.8)	2 (11.1)
E545K	0 (0.0)	4 (22.2)
H1047X	3 (18.8)	8 (44.4)
Other	4 (25.0)	3 (16.7)
Estrogen receptors expression (%), median (IQR) ^f^	90 (70–100)	95 (90–100)
Progesterone receptors expression (%), median (IQR) ^f^	80 (15–100)	60 (30–90)
Ki-67, median (IQR) ^g^	23 (12–38)	25 (11–35)
Categorized Ki-67 (%) ^g^		
Low (<14%)	5 (29.4)	5 (26.3)
Intermediate (14–19%)	0 (0.0)	3 (15.8)
High (≥20%)	12 (70.6)	11 (57.9)

Data are presented as n (%) unless otherwise stated. Metastasis sites’ categories are not mutually exclusive. Abbreviations: IQR, interquartile range; Ki-67, proliferation index; PIK3CA, phosphatidylinositol-4,5-bisphosphate 3-kinase catalytic subunit alpha; E542K/E545K, glutamate-to-lysine substitutions at codons 542/545; H1047X, any histidine 1047 substitution (e.g., H1047R, H1047L). ^a^ Available data for 21 patients in experimental and 20 in control arm. ^b^ Available data for 16 patients in experimental and 14 in control arm. ^c^ Available data for 16 patients in experimental and 20 in control arm. ^d^ Available data for 20 patients in experimental and 19 in control arm. ^e^ Available data for 16 patients in experimental and 18 in control arm. ^f^ Available data for 19 patients in experimental and 19 in control arm. ^g^ Available data for 17 patients in experimental and 19 in control arm.

**Table 3:** Exposure-adjusted grade 3–4 hyperglycemia by treatment arm (safety set, mITT).

	n	Events (G3–4)	Person-Years	EAIR (/100 PY)	95% CI
From Day 1					
Control	20	14	1.9	742	(406–1245)
Experimental	21	11	2.9	378	(189–677)
From Day 2					
Control	20	11	2.6	428	(214–766)
Experimental	21	9	3.3	269	(123–511)

Primary analysis “From Day 1”; sensitivity analysis “From Day 2” excludes Day 1 person-time and Day 1 events. Abbreviations: EAIR, exposure-adjusted incidence rate per 100 person-years; CI, exact Poisson 95% confidence interval; Events: first G3–4 hyperglycemia per participant within the window, person-years include the event day.

## Data Availability

The data and Stata code are available from the corresponding author upon request. The trial protocol and statistical analysis plan are also available from the corresponding author upon reasonable request.
